# Management of COVID-19 crisis; focus on immunization, vaccination, and safe re-locating of health care providers

**DOI:** 10.1016/j.amsu.2020.11.057

**Published:** 2020-11-25

**Authors:** Ibrahim Al-Faouri, Razan Haddad, Wail Hayagneh, Nail Obeidat, Mohammad Al-Ghazo, Nasr Alrabadi

**Affiliations:** a Department of Community and Mental Health Nursing, Faculty of Nursing, Jordan University of Science and Technology, Irbid, 22110, Jordan; b Department of Pharmaceutical Technology, Faculty of Pharmacy, Jordan University of Science and Technology, Irbid, 22110, Jordan; c Department of Pediatrics and Neonatology, Faculty of Medicine, Jordan University of Science and Technology, Irbid, 22110, Jordan; d Department of Obstetrics and Gynecology, Faculty of Medicine, Jordan University of Science and Technology, Irbid, 22110, Jordan; e Department of Surgery and Urology, Faculty of Medicine, Jordan University of Science and Technology, Irbid, 22110, Jordan; f Department of Pharmacology, Faculty of Medicine, Jordan University of Science and Technology, Irbid, 22110, Jordan

**Keywords:** COVID-19, Pandemic, Vaccines, Immunization, Health-care providers (HCPs)

The COVID-19 pandemic is currently considered the most serious threat to health-care providers (HCPs). This crisis is challenging the health-care systems for being able to preserve the physicians, nurses, and other HCPs from getting the infection and from being the source for its dissemination to the public. After more than a year of the beginning of the viral spread all over the world and ultimately being extensively spread in our communities, different perspectives, attitudes, and strategies may be needed. In this report, we will highlight a few ideas and thoughts that may help in improving and updating our strategies in facing this crisis especially with the limited resources and the possible delays in delivering the vaccines worldwide. Less fear of the COVID-19 disease among the infected HCPs and re-locating them to the frontlines of health-care systems, screening for the levels of immunity against COVID-19 at least among HCPs and patients with high risks, and accordingly, determining those who can benefit more from the limited available vaccines may become critical in facing the crisis in the near future. Drug companies, politicians, and regulatory bodies should be conservative about the production and the wide use of the vaccines without more proper validations. Those validations can reduce the unnecessary use of the vaccines in wealthy countries and can preserve more vaccines for equity distribution to the people at risk in the developing countries.

At the beginning of the crisis, the focus of the health care systems, including ours in Jordan, was to prevent or limit the entrance of the infected people through our borders. This was justifiable especially with the limited available information (at that time) about the virus, its transmission, complications/risks, and treatments. A state of emergency and the lockdown laws, including the closure of the borders, were activated to restrict the community spread of the virus. With time, the virus entered the country, through shipments/shipmen on the borders, and spread in the community. The lockdown law became less sufficient and possibly may cause harm to the economy and the citizens more than the pandemic itself.

Thereafter, the focus was shifted to slow down the community spread of the virus and to control it within the capacity of the health-care systems. On the other hand, there was a tremendous fear among physicians, nurses, and other health-care providers (HCPs) of being infected with the virus. As well, the patients themselves were extremely worried about getting the infection from the HCPs who possibly have a higher chance of contacting infected patients with the COVID-19. This fear was also reflected in the decisions of the policymakers and the focus was on finding strategies to protect the health-care system by preserving and not continuously losing the HCPs who are infected with the virus. Therefore, this extensively affected the health services provided to the patients in general and cause the closure of many and possibly all the outpatients’ clinics and restricted the health services to limited numbers of emergency cases.

With time, increasing numbers of nurses and physicians got infected with the virus. Currently, the community spread of the virus is relatively high in Jordan with more than 5000 weekly confirmed new cases per Million citizens. The anticipated actual number of newly infected cases is higher than that giving the high percentage of asymptomatic cases and the limited number of PCR tests that can be routinely conducted routinely. As well, many drug companies announced the success of phase 3 clinical trials to develop vaccines that may protect against this virus. However, the amount of those vaccines may not be enough for the whole population worldwide and limited resources may restrict their distribution in poor or developing countries. As well, many experts are suspicious about the effectiveness of those vaccines. Therefore, this urge for applying different strategies and possibly looking at facing the COVID-19 pandemic from different aspects/angles that may be more suitable for those countries.

Few reports indicated the possibility of COVID-19 re-infection. However, they are limited to a few cases of a persistent positive PCR test for confirmed infected patients after being negative and the announcement of the patient's cure. The Center for Disease Control and Prevention (CDC) announced clearly that the occurrence of re-infection needs more measurements and valid studies to be proven [1]. Many assumptions can explain this phenomenon and negate the re-infection theory [2]. Away from this discussion, which is out of the scope of this report, very few cases of possible re-infections were reported, and this could indicate at least that the re-infection is rare or, alternatively, is mild and possibly asymptomatic. In any way, this can in turn indicates that the infected people with the COVID-19 are relatively protected. On the other hand, if this is not true, then, this can challenge the value of the vaccination for the COVID-19. If the actual infection of the COVID-19 cannot protect the patients, then, it is problematic to argue that the vaccine is protective as well.

In this regard, more studies should be conducted to evaluate the acquired immunity after the infection with the COVID-19 and the sufficiency of this natural immunization (the effectiveness and for how long it can last) in protecting against the COVID-19 disease. This includes a serological screening for specific antibodies and different molecular pathways that possibly can determine the immunity against this virus. Concomitantly, the drug companies and the developers of the vaccines should in turn put more effort into clarifying these points on their studied vaccines. Rushing those vaccines to the market without clarifying those points will jeopardize the validity of using them and if proven not effective, high costs will be put on the health care systems with losing the trust of the vaccination process in general. This, in turn, can put more challenges on persuading the public to use any vaccines in the future, including vaccines for diseases other than the COVID-19.

On the other hand, with the community spread of the COVID-19 disease and the high numbers of HCPs who got infected, the idea of re-locating the infected HCPs towards the frontlines of the health-care systems may be valid. While preserving the non-infected HCPs to work on the floors and only working with the patients after testing the suspected cases for COVID-19 infection. Using this strategy may be more effective or at least equally effective to the use of the vaccines among the HCPs. As well, all HCPs could be tested to evaluate their immunization status, then, those with sufficient amounts or measurements of COVID-19 antibodies can be assumed to have a previous asymptomatic infection with COVID-19 and can also be re-located to the frontlines of the health care systems.

Screening for the antibodies that indicate the COVID-19 immunity and for how long it can last in the body of the previously infected people can also indicate the longevity of the protecting immunity and the sufficiency of the vaccination process and for how long it can last. The HCPs are the best group of people who can give indication or answers to those questions if being tested properly [3]. As they are the group with the higher risk for re-infection (if possible) and the first group that may be getting infected and immunized with the virus. This is critical in understanding how to deal with high-risk patients and in preserving the vaccines for those people who need it (highly-risk and non-immunized people).

To conclude, with the increasing numbers of infected HCPs, fear of the COVID-19 disease among them should be lessened and new strategies of re-locating them toward the frontlines of the health-care systems may be valid and sufficiently similar to the protection expected by the vaccination process, if not more. Screening for the immunization status and its longevity is critical in directing the limited vaccines to the high-risk non-immunized people who can benefit most from it. Drug companies, politicians, and regulatory bodies should be conservative about the production and the wide use of the vaccines without more proper validation. Those validations can reduce the unnecessary use of the vaccines in wealthy countries and can preserve more vaccines for equity distribution to the people at risk in the developing countries.

## Conflict of interest statement

The authors declared no conflict of interest.

## Funding

Not Applicable.
